# Carbon monoxide and risk of outpatient visits due to cause-specific diseases: a time-series study in Yichang, China

**DOI:** 10.1186/s12940-019-0477-3

**Published:** 2019-04-23

**Authors:** Yu Wang, Chengye Yao, Chengzhong Xu, Xinying Zeng, Maigeng Zhou, Yun Lin, Pei Zhang, Peng Yin

**Affiliations:** 1 Department of Anesthesiology, Institute of Anesthesia and Critical Care Medicine, Union Hospital, Tongji Medical College, Huazhong University of Science and Technology, Wuhan, 430022 China; 2 Department of Neurology, Union Hospital, Tongji Medical College, Huazhong University of Science and Technology, Wuhan, 430022 China; 3 Yichang Center for Disease Control and Prevention, 3 Dalian Road, Yichang, 443005 China; 4 National Center for Chronic and Noncommunicable Disease Control and Prevention, Chinese Center for Disease Control and Prevention, 27 Nanwei Road, Xicheng District, Beijing, 100050 China

**Keywords:** Air pollution, Carbon monoxide, Outpatient visit, Health effect, Time-series study

## Abstract

**Background:**

Previous studies showed inconsistent results on risk of increased outpatient visits for cause-specific diseases associated with ambient carbon monoxide (CO).

**Methods:**

Daily data for CO exposure and outpatient visits for all-causes and five specific diseases in Yichang, China from 1st January 2016 to 31st December 2017 were collected. Generalised additive models with different lag structures were used to examine the short-term effects of ambient CO on outpatient visits. Potential effect modifications by age, sex and season were examined.

**Results:**

A total of 5,408,021 outpatient visits were recorded. We found positive and statistically significant associations between CO and outpatient visits for multiple outcomes and all the estimated risks increased with longer moving average lags. An increase of 1 mg/m^3^ of CO at lag06 (a moving average of lag0 to lag6), was associated with 24.67% (95%CI: 14.48, 34.85%), 21.79% (95%CI: 12.24, 31.35%), 39.30% (95%CI: 25.67, 52.92%), 25.83% (95%CI: 13.91, 37.74%) and 19.04% (95%CI: 8.39, 29.68%) increase in daily outpatient visits for all-cause, respiratory, cardiovascular, genitourinary and gastrointestinal diseases respectively. The associations for all disease categories except for genitourinary diseases were statistically significant and stronger in warm seasons than cool seasons.

**Conclusion:**

Our analyses provide evidences that the CO increased the total and cause-specific outpatient visits and strengthen the rationale for further reduction of CO pollution levels in Yichang. Ambient CO exerted adverse effect on respiratory, cardiovascular, genitourinary, gastrointestinal and neuropsychiatric diseases especially in the warm seasons.

**Electronic supplementary material:**

The online version of this article (10.1186/s12940-019-0477-3) contains supplementary material, which is available to authorized users.

## Introduction

Carbon monoxide (CO) is an air pollutant primarily from traffic or industry in most urban communities. The human exposure studies have well documented acute CO poisoning at high concentrations [1]. As for environmentally relevant CO, recent epidemiological studies have found that ambient CO has significant adverse effects on public health worldwide [2–4]. The population-based studies from 126 United States urban counties showed the positive effects of ambient CO on cardiovascular disease (CVD) hospital admissions [3]. An European study conducted in 6 Italian cities showed significant and positive associations between CO and emergency room visits for acute respiratory diseases (RED) [4]. However, some recent experimental and clinical studies suggested that low levels of exogenous CO may have therapeutic effects under certain circumstances [5, 6], and population-based studies in China generated similar findings that environmentally relevant CO exposure reduced risk of hospital admissions for respiratory tract infections, stroke and chronic obstructive pulmonary diseases [7–9]. In a study of 10 United States cities, it was also indicated that 1-ppm increase of CO was associated with a 0.7% decrease in daily mortality [10].

Furthermore, because ambient CO primarily results from traffic or industry in urban communities, risks associated with CO may be confounded or modified by other traffic-related air pollutants, such as nitrogen dioxide (NO_2_), sulfur dioxide (SO_2_), ozone (O_3_) and fine particles (particulate matter with aerodynamic diameter ≤ 10 μm [PM_10_] or  ≤ 2.5 μm [PM_2.5_]). The experimental and clinical studies can provide useful scientific evidence but typically involve exposure to CO alone [3]. The lack of co-pollutant models has contributed to the inability to disentangle the effects attributed to ambient CO from those of the larger complex air pollution mix.

Many studies have reported the association between ambient CO and cardiorespiratory diseases [11–13]. However, other common diseases such as neuropsychiatric (NPD), genitourinary (GUD) and gastrointestinal diseases (GID) were rarely examined. In recent years, some studies showed the associations between ambient CO and other diseases besides cardiorespiratory systems [4, 14], which may be important to consider when the policies regarding CO standards and guidelines are evaluated. Therefore, all of these together point towards a need for a comprehensive understanding of the health effects for various body systems induced by ambient CO exposure, especially at low concentrations of CO.

As the largest low- and middle-income countries, China is experiencing one of the worst air pollution problems in the world. However, in Yichang, a city located in Hubei province in central China with 4.2 million people, outdoor CO levels are low (daily average of 1.07 mg/m^3^) and well below the World Health Organization (WHO) guideline of 10 mg/m^3^. Little research has been done on the potential health effects in humans from current ambient exposure to generally low CO levels, especially in China. Research has been focused on air pollution associated mortality in China and there has been limited research on the association between air pollution and morbidity, such as outpatient visits for specific diseases mainly due to limited access to high-quality hospital data. Yichang, however, is one of only a handful of cities in China where data from all hospitals are collected in a systematic manner to a single database and therefore an ideal city for researching the effects of air pollution on outpatient visits in China.

In the current study, a time-series analysis was performed to evaluate the short effects of exposure to ambient CO on outpatient visits for total causes and RED, CVD, NPD, GUD and GID. We addressed key scientific questions about associations of CO at low levels with cause-specific outpatient categories, possible confounding by co-pollutants in the urban air pollution mixture and effects modifications for age, sex and season.

## Materials and methods

### Data collection

#### Hospital outpatient data

Health data was collected from all health organizations in Yichang, from district to city level health facilities, and stored on a cloud server which is run by Yichang Center for Disease Control and Prevention (CDC). Daily cause-specific outpatient data for eight of the largest hospitals in the city of Yichang (accounting for 96% of all outpatient visits in the city) were obtained from the Big Data Centre, covering the period from 1st January 2016 to 31st December 2017. Anonymised outpatient visits records were extracted according to age, gender, the date of visit and International Classification of Diseases, Tenth Revision (ICD-10). All of the outpatient visits were further classified by the ICD-10, for total causes: A00-Z99, CVD: I00-I99, RED: J00-J99, GUD: N00-N99, GID: K00-K93, NPD: F00-G99. For further analyses, we also divided the total causes outpatient visits to different age groups (0 ≤ age < 6, 6 ≤ age < 65 and age ≥ 65), gender groups (male and female). The whole year was divided into two seasons, warm season (April to September) and cold season (January to March and October to December), according to the seasonal characteristics of Yichang. Ethics approval and consent from individuals were not required, as only aggregated non-identifiable data were used in this study.

#### Air pollution and weather factors data

Data on concentration of CO, were obtained from Yichang Municipal Bureau of Environmental Protection from 1st January 2016 to 31st December 2017. The bureau was responsible for the monitor stations which provided hourly air pollution data to the Big Data Centre of Yichang CDC. The data for pollutants was an average of the daily readings from each of the 14 air quality monitoring stations. We also included measurements of PM_2.5_, PM_10_, SO_2_, NO_2_, and O_3_ for adjustment in multi-pollutant models. Missing data were identified for air pollutant variables for 2 days out of the two-year period. As 2 days only accounts for 0.27% of the total number of days in the study period, dates with missing values were excluded from the analysis. In addition, we got the meteorological variables contained daily (24-h) average temperature and relative humidity (RH) from the Big Data Centre in Yichang to allow for adjustment of weather factors on outpatient visits.

### Statistical analysis

Outpatient visits were linked with air pollutant concentrations by date. Generalized additive models (GAM) were used to investigate the associations between daily concentrations of CO and daily counts of outpatient visits for total causes, CVD, RED, GUD, GID and NPD. Quasi-Poisson regression was used in the model because outpatient visits tended to display an over-dispersed poisson distribution. Specifically, we used 5–10 degrees of freedom (df) per year for time trend. When the df was 7, the absolute magnitude of the partial autocorrelation function was lowest, so the basic model was regarded as adequate [15] and a cubic spline function with 7 df per year was applied to calendar time to account for unmeasured long-term and seasonal trends. Cubic spline functions were also applied to current-day temperature (6 df) and humidity (3 df), to allow for adjustment of potential meteorological confounding factors [16]. Day of the week and season were also included in the basic model to adjust for the day effect on outpatient visits within a week and season effect within a year. Public holidays were introduced as a dummy variable to adjust for the holiday effects.

After we constructed the basic models, we introduced the CO variable to create a single-pollutant model to estimate the association with total causes outpatient visits, and then separately for different diseases categories. We revealed the lag effects with various lag structures—from the days of outpatient visit (lag 0) up to seven lag days (lag 7). In addition, the models included the moving averages as averages of the exposure lags to avoid underestimating the effect of pollutants measured by single-day lag models [17]. For example, the 2-day moving average (lag 01) was concentration computed as the means of lag 0 and lag 1 days.

Previous literatures [18, 19] has suggested there were effect modifications for age, gender and season when investigating the effects of air pollution on hospital visits. Therefore, additional analyses were conducted to explore the potential modifications by age, gender and season subgroups. We evaluated the statistical significance for the differences in different age groups, gender and season [14]. To examine the stability of CO on outpatient visits, multi-pollutant analyses were performed to adjust for the other pollutants included NO_2_, O_3_, SO_2_, PM_2.5_ and PM_10_, using the same parameter settings as in the main model. Finally, exposure-response (E-R) curves using the same models at lag 06 additionally using a spline function to model the exposure variable, were created to assess CO concentrations against cause-specific outpatient visits.

Effect estimates were described as percent changes and 95% CIs in daily outpatient visits for total causes and different diseases per 1 mg/m^3^ increase in CO. The statistical tests were two-sided, and *P*-values< 0.05 were considered statistically significant. All analyses were performed using the SAS (version 9.4; SAS Institute Inc.) and MGCV package in the R software (R 3.5.0).

## Results

Table 1 summarizes descriptive statistics of this study on outpatient data, air pollutants and weather factors in Yichang from 1st January 2016 to 31st December 2017. A total of 5,408,021 cases of outpatient visits occurred over the two-year study period (731 days), with a daily mean of 7418 cases. Females and the patients between 6 and 64 years of age accounted for 58.7 and 70.4%, respectively. Outpatient visits were slightly higher in cool seasons (50.5%) than in warm seasons (49.5%). Daily concentrations of CO were low during the study period, with a daily average of 1.07 mg/m^3^ and a maximum of 2.63 mg/m^3^ in Yichang (The WHO air quality guideline for CO is 10 mg/m^3^). The mean of daily temperature and relative humidity were 17 °C and 77%, respectively. Table 2 shows the daily concentration of CO was moderately and positively correlated with PM_10_, PM_2.5_, NO_2_ and SO_2_ (Pearson correlation coefficient r = 0.42–0.72), and negatively correlated with O_3_ and temperature. The daily average of relative humidity were not correlated with CO (r < 0.1).

**Table 1:** Summary statistics of outpatient visits, air pollutants and meteorological factors in Yichang, China

Variables	Number	Daily mean ± SD	Min	Median	Max
Outpatient visits					
Total(ICD:A00-Z99)	5,408,021	7418 ± 2376	889	7369	13,770
Gender					
Male	2,230,860	3060 ± 921	471	3037	5473
Female	3,177,161	4358 ± 1465	472	4328	8437
Age (year)					
0~5	657,340	901 ± 285	116	871	1727
6~64	3,808,042	5223 ± 1672	581	5167	9751
65~	942,639	1293 ± 570	132	1323	2982
Season					
warm	2,676,916	7313 ± 2309	1404	7100	12,558
cold	2,731,105	7523 ± 2439	943	7504	13,770
CVD(ICD:I00-I99)	577,721	792 ± 337	47	811	1826
RED(ICD:J00-J99)	901,387	1236 ± 383	205	1225	2655
NPD(ICD:F00-G99)	248,344	340 ± 125	23	335	772
GUD(ICD:N00-N99)	567,368	778 ± 289	48	800	1752
GID(ICD:K00-K93)	432,391	593 ± 208	65	600	1142
Air pollutant (24-h Average)					
CO (mg/m^3^)		1.07 ± 0.33	0.4	1.02	2.63
PM_2.5_ (μg/m^3^)		59.49 ± 42.32	4.5	48.17	263.12
PM_10_ (μg/m^3^)		95.26 ± 52.27	10.08	84.54	340.5
NO_2_ (μg/m^3^)		45.05 ± 21.21	13.21	33.33	81.21
SO_2_ (μg/m^3^)		12.27 ± 4.88	4.33	11.38	45.64
O_3_ (μg/m^3^)		45.05 ± 21.21	10.67	42.62	124.29
Meteorological factors(24-h Average)					
Temperature (°C)		16.85 ± 8.19	−1.1	17.15	32.6
RH (%)		76.82 ± 14.29	31	77.3	99

Abbreviation: *CVD* cardiovascular diseases, *RED* respiratory diseases, *NPD* neuropsychiatric diseases, *GUD* genitourinary diseases, *GID* gastrointestinal diseases, *RH* relative humidity, *SD* standard deviation, *min* minimal, *max* maximal

**Table 2:** Pearson correlation coefficients for meteorology factors and air pollutants

	PM_2.5_	PM_10_	CO	NO_2_	SO_2_	O_3_	Temperature	RH
PM_2.5_	1.00							
PM_10_	0.94	1.00						
CO	0.77	0.67	1.00					
NO_2_	0.70	0.72	0.55	1.00				
O_3_	−0.40	−0.29	−0.46	−0.38	1.00			
SO_2_	0.61	0.64	0.42	0.51	−0.24	1.00		
Temperature	−0.62	−0.53	−0.53	−0.49	0.57	−0.54	1.00	
RH	−0.18	−0.30	0.05	−0.33	− 0.16	− 0.29	0.15	1.00

Abbreviation: *RH* relative humidity

**Fig. 1:**
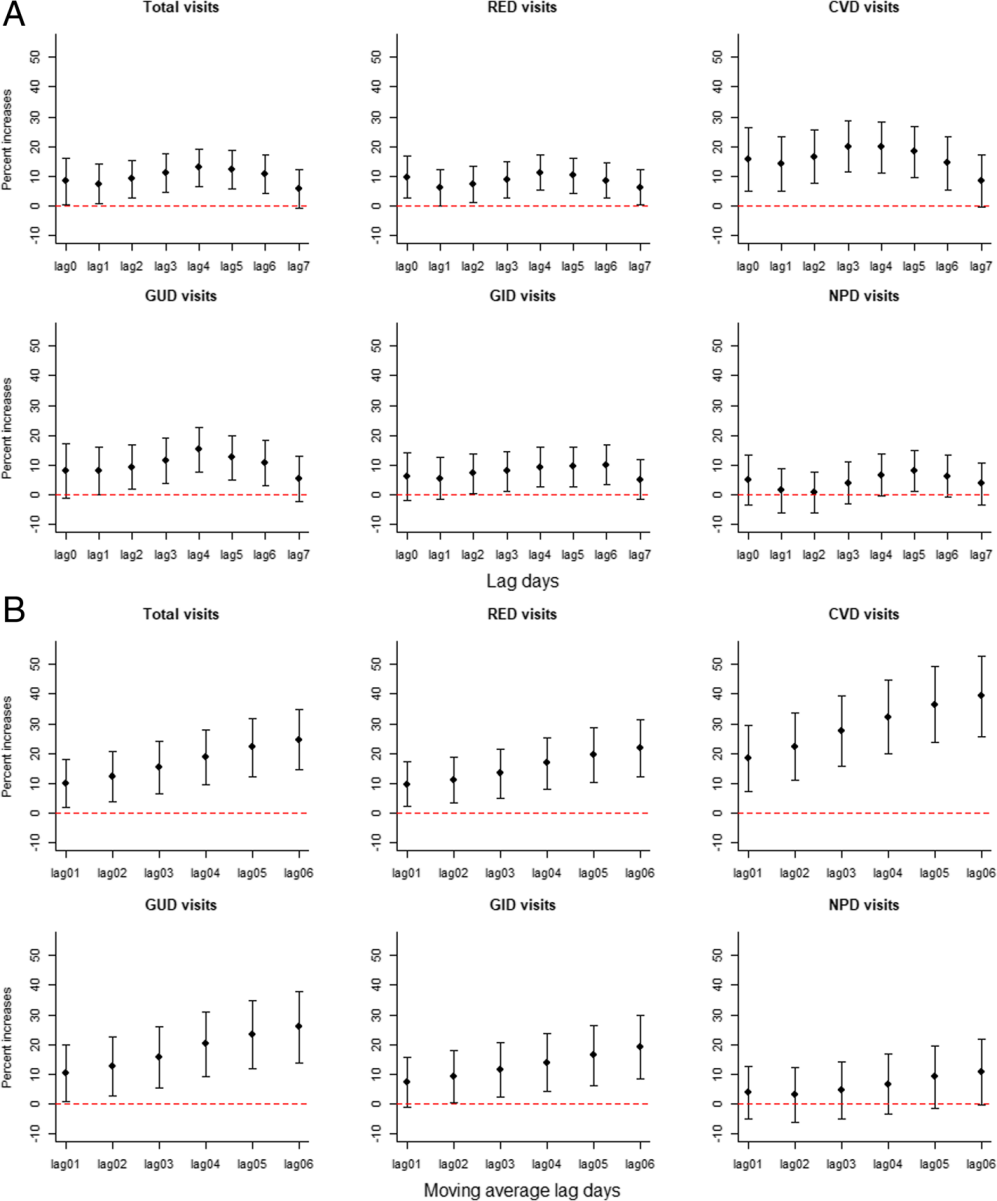
Percent changes of cause-specific outpatient visits associated with 1 mg/m^3^ increments in CO. Abbreviation: *RED* respiratory diseases, *CVD* cardiovascular diseases, *GUD* genitourinary diseases, *GID* gastrointestinal diseases, *NPD* neuropsychiatric diseases. Note: The X-axis is the lag days from lag0 to lag7 (**a**), and the moving average lag days from lag 01 to lag 06 (**b**); The Y-axis is the percent increases of daily outpatient visits; the points indicate central estimates; Bars, 95% confidence intervals

Figure 1 presents the percent changes and 95% CIs of outpatient visits for total causes, RED, CVD, GUD, GID and NPD associated with 1 mg/m^3^ increments in CO at different single lags and moving average lag days. The analyzed outpatient categories for total, RED, CVD, GUD and GID, with the exception of NPD, were statistically significant and positively associated with the most of the lag periods concentrations of ambient CO, while NPD showed the only statistically significant association at lag 5. The associations were not statistically significant for all outcomes at lag 7. For moving averages lag days from lag 01 to lag 06, the associations for all of the outpatient categories except NPD were statistically significant and positive, and all the estimated risks increased as the average of longer lags were considered. The percent increases were strongest at lag 06 days for all outpatient categories, and a 1-mg/m^3^ increase in concentration of CO was associated with increments of 24.67% (14.48, 34.85%), 21.79% (12.24, 31.35%), 39.30% (25.67, 52.92%), 25.83% (13.91, 37.74%), 19.04% (8.39, 29.68%) in daily outpatient visits for total causes, RED, CVD, GUD and GID, respectively.

Table 3 summarizes the results for possible effect of modification by age, gender, and season at lag 06 days. For age and gender subgroups, the percent increases of outpatient visits due to total causes, RED, CVD, GUD and GID were statistically significant and positive, except CVD and GID who aged 0 to 5 years. The percent increases between CO and outpatient visits varied by gender and age subgroups, but were similar with the overall patients in different outpatient categories. The associations appeared to be more notable for the old patients (age ≥ 65) except for GUD outpatient visits induced by CO, although their between-age difference was statistically insignificant. The association between ambient CO concentration and number of NPD outpatient visits was only statistically significant for the old patients (age ≥ 65). We found CO were related to increased risk of total-causes and CVD outpatient visits in both warm and cool seasons while the associations became insignificant for other outpatient categories in the cool season. The associations for all of the outpatient categories except GUD were statistically significant and stronger in the warm seasons than in cool seasons and the difference was statistically significant for outpatient visits due to total causes, CVD and NPD.

**Table 3:** Associations of daily outpatient visits by age, sex and season with ambient CO

Subgroups	Total visits	RED visits	CVD visits	GUD visits	GID visits	NPD visits
Overall patients^a^	**24.67(14.48,34.85)**	**21.79(12.24,31.35)**	**39.30(25.67,52.92)**	**25.83(13.91,37.74)**	**19.04(8.39,29.68)**	10.84(−0.22,21.90)
Gender						
Male	**23.39(13.40,33.38)**	**22.31(12.44,32.18)**	**38.21(24.18,52.24)**	**22.85(9.43,36.27)**	**19.25(8.37,30.13)**	11.35(−0.28,22.99)
Female	**25.58(15.10,36.06)**	**21.24(11.58,30.90)**	**40.54(26.85,54.22)**	**26.55(14.22,38.89)**	**18.85(7.63,30.07)**	10.41(−1.27,22.10)
Age (year)						
0~5	**19.67(9.67,29.68)**	**21.74(11.46,32.02)**	20.26(−4.73,45.25)	**35.68(6.02,65.35)**	11.87(−1.37,25.12)	7.86(−26.61,42.32)
6~64	**24.00(13.52,34.48)**	**21.48(11.16,31.81)**	**36.18(23.34,49.03)**	**26.18(14.04,38.32)**	**17.90(6.68,29.12)**	9.51(−1.71,20.72)
65~	**30.78(18.43,43.14)**	**23.61(11.58,35.64)**	**42.99(27.38,58.60)**	**20.96(6.02,35.91)**	**29.54(17.03,42.06)**	**15.00(1.18,28.81)**
Season						
Warm	41.09(22.04,60.15)*	**27.83(7.92,47.74)**	68.04(40.48,95.61)*	22.79(−0.72,46.29)	**21.98(0.32,43.64)**	25.80(8.14,43.46)*
Cool	16.13(2.52,29.74)*	11.61(−0.62,23.85)	38.57(20.14,56.99)*	14.08(−1.42,29.58)	9.73(−4.19,23.64)	−4.18(−20.27,11.91)*

Abbreviation: *RED* respiratory diseases, *CVD* cardiovascular diseases, *GUD* genitourinary diseases, *GID* gastrointestinal diseases, *NPD* neuropsychiatric diseases

Note: Results was estimated percent increases and its corresponding 95% confidence intervals with 1 mg/m^3^ increase in CO at lag06 (lag06 was concentration computed as the means of the same and previous 6 days);

^a^Overall patients means all of the patients in different outpatient categories; The statistically significant estimates are highlighted in bold

*Statistically significant for between-group difference (*P* < 0.05)

**Fig. 2:**
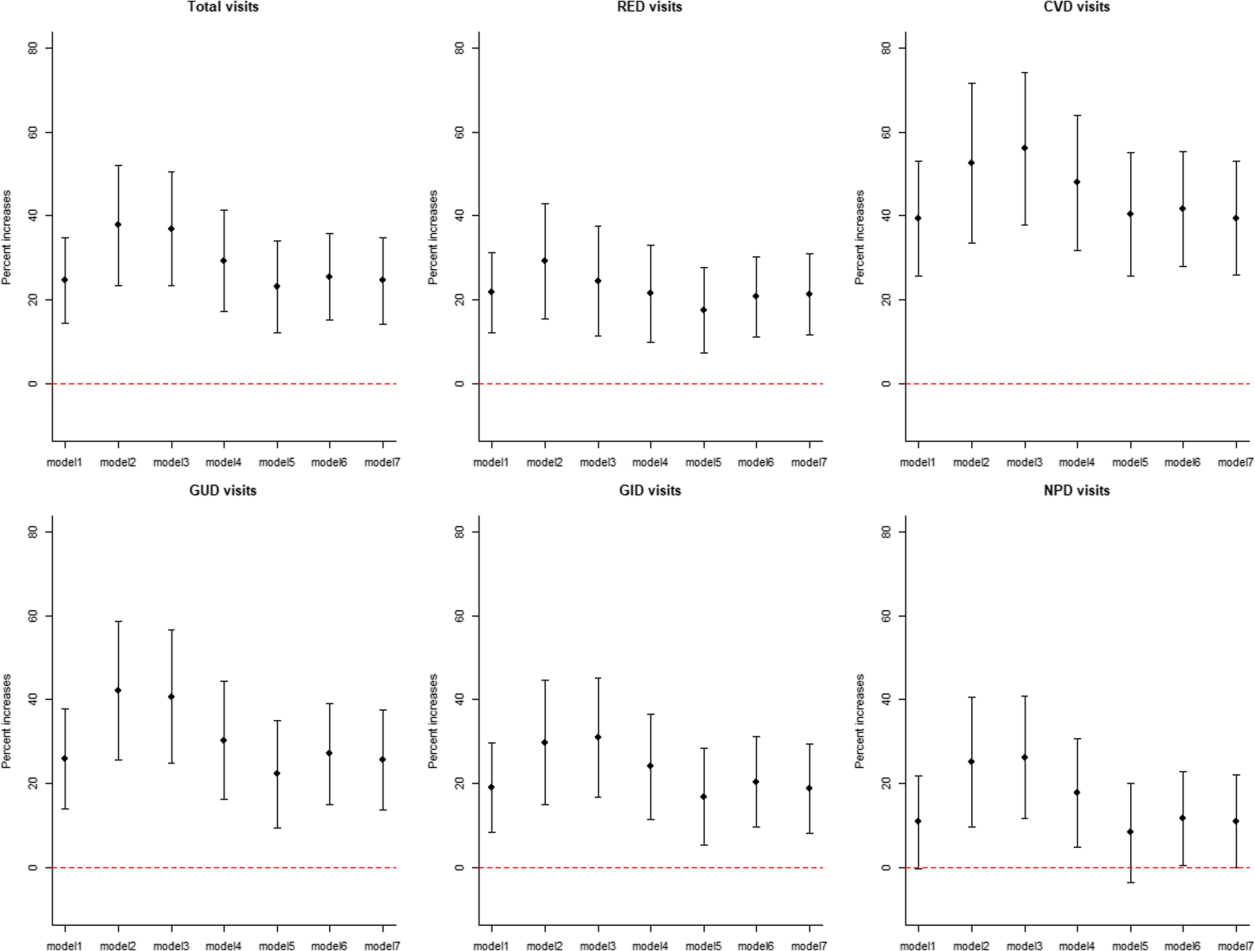
Percent increases of outpatient visits associated with 1-mg/m^3^ increase in CO in multi-pollutant models. Abbreviation: *RED* respiratory diseases, *CVD* cardiovascular diseases, *GUD* genitourinary diseases, *GID* gastrointestinal diseases, *NPD* neuropsychiatric diseases. Note: All the models were constructed for pollutant at lag06. Model1: Single-pollutant model for CO at lag06; model2: six-pollutants model including PM_2.5_, PM_10_, NO_2_, SO_2_, O_3_ and CO; model3: two-pollutant model adjusted for PM_2.5_; model4: two-pollutant model adjusted for PM_10_; two-pollutant model adjusted for NO_2_; two-pollutant model adjusted for SO_2_; two-pollutant model adjusted for O_3_. The points indicate central estimates; Bars, 95% confidence intervals

Figure 2 shows the results for outpatient categories of multiple outcomes with a 1-mg/m^3^ increase in CO at lag 06 in multi-pollutants models. The associations of CO and outpatient visits for total causes, RED, CVD, GUD, GID and NPD were still stable and statistically significant after the adjustments of the five air pollutants (PM_2.5_, PM_10_, SO_2_, NO_2_ and O_3_), and were strengthened, especially with particulate matter adjustments. For example, the percent increases for total-causes outpatient visits at lag 06 days were 24.67% (14.48, 34.85%) in single-model contained CO, and the observation was correlated with a 36.85% (23.26, 50.45%) and 29.24% (17.20,41.27%) visits rise in two-pollutant models adjusted for PM_2.5_ and PM_10_, respectively. The effects by other pollutants in the multi-pollutant models are displayed in Additional file 1: Table S1.

**Fig. 3:**
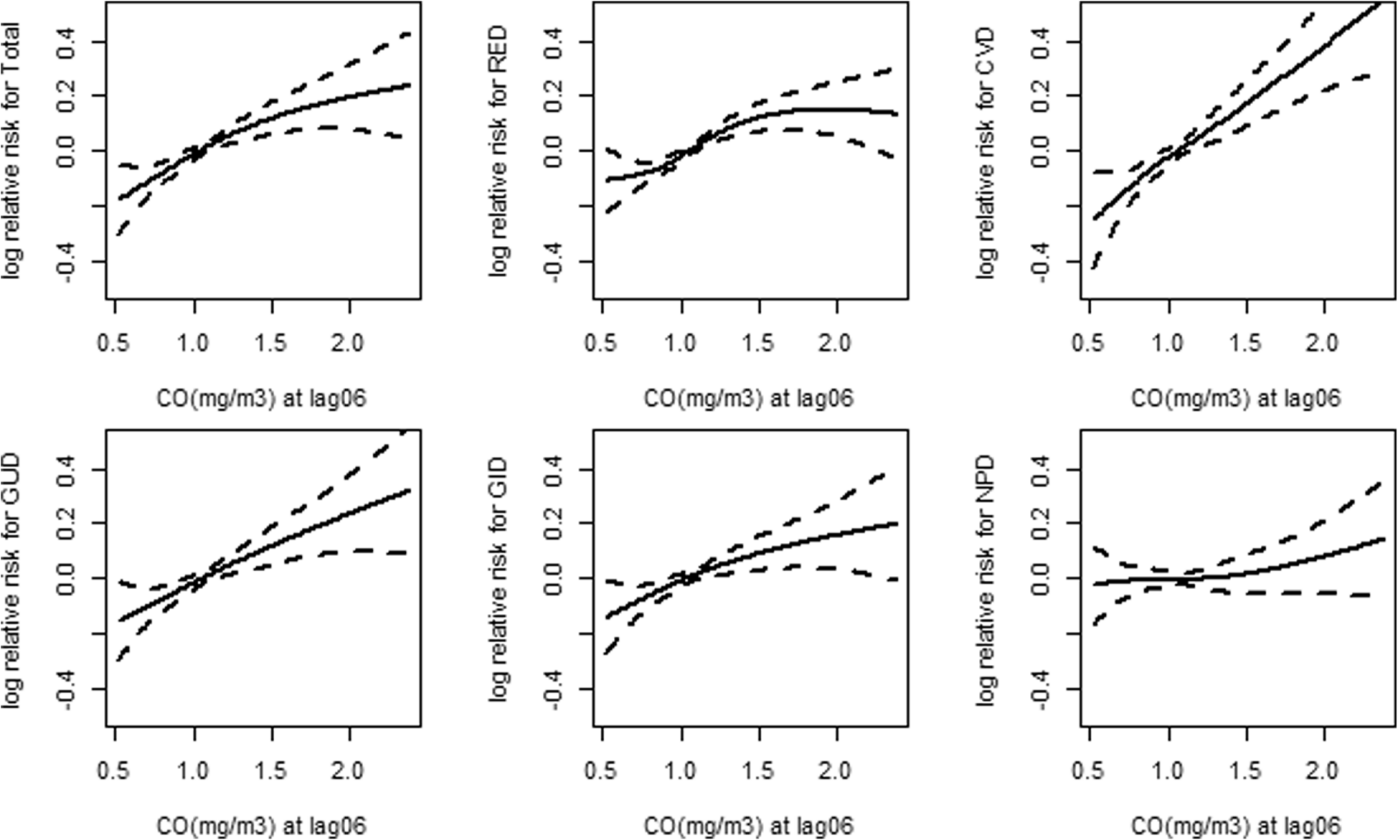
The exposure-response relationship curves between CO concentrations at lag06 and daily cause-specific outpatient visits. Abbreviation: *RED* respiratory diseases, *CVD* cardiovascular diseases, *GUD* genitourinary diseases, *GID* gastrointestinal diseases, *NPD* neuropsychiatric diseases. Note: The X-axis is the concentrations of air pollutants on the lag06 days (lag06 was concentration computed as the means of the same and previous 6 days); The Y-axis is the log relative risk; The solid line represents the predicted log relative risk, and the dotted lines represent the 95% CI

Figure 3 shows the exposure-response (E-R) associations between CO concentrations at lag 06 days and outpatient visits for total causes, RED, CVD, GUD, GID and NPD. The E–R relationships for CO with CVD and GUD outpatient visits were almost linear, showing no thresholds for their associations. For the curve of CO with RED outpatient visits, we observed a relatively flat slope at concentrations below 1 mg/m^3^, and then a drastic increase at concentrations 1 mg/m^3^ to 1.5 mg/m^3^. The E–R curve of CO with total and GID outpatient visits showed a moderately positive association. The curve of CO with NPD outpatient visits showed a relatively flat slope at concentration 0.5 mg/m^3^ to about 2 mg/m^3^.

## Discussion

This study examined the acute effect of ambient CO on outpatient visits for total causes, RED, CVD, GUD, GID and NPD in Yichang, China. We found positive associations between CO and outpatient visits for multiple outcomes (total causes, RED, CVD, GUD, GID and NPD outpatient visits) on different lag days. The effect on outpatient visits was immediate and can persist for up to seven days, and all the estimated risks increased as the moving average of longer lag days were considered. To the best of our knowledge, this is the first multi-outcome study for ambient CO in low- and middle-income countries, to examine the relationship between CO and outpatient visits for total causes, RED, CVD, GUD, GID and NPD.

Our findings about the adverse effects of CO for outpatient visits of different diseases were generally in consistent with previous studies [20–24]. Most articles indicated that ambient CO was related to increased hospital visits or admissions for RED and CVD. A study in Spokane, Washington showed that ambient CO exhibited a positive association with RED emergency room visits and showed larger effects at longer lag days for CO [21]. A 1 ppm increase in the 3-day lag of CO was associated with a 1.03-fold increase in respiratory emergency room visits [21] which is very close to our results for RED (1.07-fold increase per ppm increase in CO at lag3). A meta-analysis showed the association between CO and emergency room visits/hospital admissions for asthma in the overall analyses (42 studies) were positive, and the pooled relative risks were 1.07-fold increase per 1 mg/m^3^ increase in CO [24] which is a little smaller than our results for all RED (1.10-fold increase per 1 mg/m^3^ increase in CO). Szyszkowicz [25] used a generalized linear mixed model found a significant association between 0.2 ppm ambient CO and emergency department visits for ischemic heart disease (5.4% [95% CI, 2.3, 8.5%]) which is bigger than our results for all CVD (2.5% [95% CI, 0.8, 4.2%] for 0.2 ppm CO). A multi-city time-series analysis in Canada [22] showed that day average concentrations of CO exhibited the positive associations with visits for myocardial infarction/angina for (2.1% [95% CI, 0.0, 4.2%]) increase in per 0.7 ppm CO and 3.8% (95% CI, 0.7, 6.9%) increase in visits for heart failure, which is smaller than our results for CVD (8.7% [95% CI, 2.7, 14.7%] for 0.7 ppm CO). As for NPD, in six cities of Canada, the percentage increase in daily hospital visits for depression was 6.9%(95% CI, 3.8, 10.1%) for CO per 0.8 ppm for same day exposure [20], almost twice our results for NPD (3.1% [95% CI, − 2.3, 8.6%] for 0.8 ppm CO). The study at 6 Italian cities showed that CO was most strongly associated with acute respiratory diseases hospital visits in 7 day average and the association between CO and gastroenteric disorders hospitalizations were also statistically significant among young children [4]. The effect size of the association for gastroenteric disorders was a 3.8% increase (95% CI, 1.0, 6.8%) per 1.1 μg/m^3^ increase in CO, which is substantially larger than our estimates. In addition, no studies examined associations between ambient CO and visits for GUD diseases and only a few studies examined pooled estimates for total outpatient visits associated with ambient CO.

However, the effects of CO on hospital visits or admissions varies considerably, especially at low levels of exposure, and conflicting results were documented. In Hong Kong, a study found a negative association between CO and risk of respiratory tract infection hospitalizations [7] and the results of some studies were negative associations between ambient CO and stroke emergency hospitalization and chronic obstructive pulmonary diseases hospitalization [8, 26]. The differences between our results and previous findings may be due to different study designs, different locations, various climate, air pollution mixture and study population. Possibly because of the higher levels of air pollution, a stronger temporal association was observed of the ambient CO concentrations on the current day for heart failure and myocardial infarction/angina hospitalizations and hospital visits [12, 13, 22]. However, the ambient CO concentrations in Yichang are mainly at a low level (the maximum daily average is 2.6 mg/m^3^), and may need more than one day to have increased health outcomes. The cumulative effect display similar temporal patterns in the multi-cities study, that the estimated risks for CO were consistently larger for the moving averages with longer lags and the strongest association for CO was at lag 06 [4]. The structure of the local health service might affect the interpretation of the observed lag effects as it may take a few days or longer to arrange an outpatient appointment rather than the time it took for CO to exert its health effects. We believe the time duration required for outpatient clinic attendances is not likely to cause any biases in this study because Yichang is a typical middle sized Chinese city where the residents normally don’t need to make arrangements for the outpatient visits and the observed lag estimates can reflect the acute effects of CO.

We found that the associations of ambient CO with all of the outpatient visits categories were stronger in warm seasons than in cool seasons which is consistent with previous research [20, 22, 23]. The stronger associations in warm seasons may be attributable to higher personal exposure to ambient air pollutants in relation to more outdoor activities and natural ventilation [27]. Besides, high temperature and strong light in warm seasons lead to enhanced photochemical reactions, resulting in stronger effects [27]. While some researchers believe that infectious diseases also show some seasonal changes, most studies do not consider this factor may lead to certain deviations [28]. Other studies suggest the biological mechanisms that elevated temperatures in warm seasons cause the thermoregulatory system to activate three major mechanisms to dissipate excess body heat (cardiovascular, respiratory activity, and sweat gland perspiration), and that activation directly or indirectly promotes more pollution enter into the body.

With adjustment for other pollutants, the association remained stable and strengthened, particularly with PM_2.5_ and PM_10_ adjustment. Given the correlations among various pollutants, it is difficult to disentangle the effects of ambient CO. However, the collinearity between CO and other ambient pollutants can be addressed for the r < 0.7 in our study (except PM_10_, r = 0.77) [29]. The shape of the E-R plot plays a role in public health assessment. In the present study, we did not observe threshold concentrations for ambient CO level with CVD and GUD outpatient visits while the risk for RED outpatient visits increased drastically at concentrations of 1 mg/m^3^ which is much lower than WHO standard. The E-R relationship between CO and outpatient visits is important for understanding the causal mechanisms of the relationship and for management of local health systems. Prior research has shown substantial heterogeneity between regions and cities [30], and it is essential to have localised E-R relationships for proper prevention.

In recent years, some researchers have begun to study the associations between ambient CO and other diseases besides respiratory and cardiovascular systems [4, 14]. In our study, we found the associations between CO and outpatient visits for NPD, GID and GUD were statistically significant and positive. For NPD, there is accumulating evidence that outdoor air pollution may have a significant impact on health and disease can adversely affect the brain and nervous system in human and animal studies [31–33]. CO, as a known neurotoxin and a potential public health threat, can cross the placenta to gain access to the fetal circulation and the developing brain [34]. Oxidative stress has been recognized as one of the main pathways by which air pollutants cause damage to cardiovascular and respiratory systems [35]. Likewise, it may also be hypothesized that air pollution may impair the nervous system through oxidative stress pathways. As for GID, although the exact mechanisms are unclear, the associations between CO and increased GID outpatient visits are somewhat biologically plausible. For example, gastroenteritis is an inflammation of the gastrointestinal tract that could be caused by infection or by adverse reaction to ingested or inhaled material so it is possible that CO are involved in the mechanism [4]. As the best of our knowledge, this is the first study to report this association of CO and GUD outpatient visits and the mechanisms underlying these effects are not well known. In light of the limited evidence in the association of CO with various diseases, verification of these associations in further studies would be necessary.

This study has several strengths. First, although previous studies have shown that increased ambient CO is associated with excess hospital visits on specific diseases, few studies were devoted to pooled estimates of ambient CO health effects using overall outpatient visits. We studied the outpatient visits for total-causes to get comprehensive estimate of health effects for CO pollution which is necessary to implement better disease control policy. Second, this study allowed us to investigate the effects of CO level on outpatient visits for RED, CVD, GUD, GID and NPD in the same setting and the same period. To the best of our knowledge, this is the first multi-outcome study for ambient CO and this could help better understand adverse effects of ambient CO to different body system. Thus, the data in our study may be important to consider when the standards and guidelines are evaluated and revised in the future. Third, many other recent studies have been based on fewer than 10,000 visits, and have examined single conditions or were restricted to specific seasons or age groups. The large sample size of 5.4 million outpatient visits in our study gives us more statistical power than many of those in other studies conducted in China. Fourth, it should be noted that risk estimates of many studies were mostly based on hospital admission data rather than on the timing of symptom onset, possibly leading to underestimation of effects [36]. Therefore, the data on outpatient visits may better reflect the acute effects of health and reduce the confounding bias. Besides, there may be many confounding factors when the patient’s condition is critical and complex in the emergency rooms and the diagnosis is prone to error, so an analysis of the outpatient visits is better to reflect health effects of ambient CO exposure. Finally, by using a time-series approach, as opposed to a case-crossover approach, this study was more effective for controlling meteorological variables, which was particularly important in this study because an entirely new location was under study [37].

Our study was subject to several limitations. First, the use of citywide average air pollution levels calculated from various monitoring stations rather than personal exposure measures will result in exposure misclassification because of the spatial distribution of ambient CO in urban areas, tending to underestimate the risk [38]. Extensive research has not been conducted on the relationship between personal exposure to CO and ambient measurements. Second, the potential misclassification caused by coding or diagnostic errors should be considered when interpreting the findings. It is not likely to be a problem in this study because all the data coming from different outpatient departments underwent stringent quality check and coding verification before they were included in the big data platform. Third, we could not obtain data on more specific subtypes for RED, CVD, GUD, GID and NPD, leading to the failure in a comprehensive analysis on air pollution and specific diseases, like cerebrovascular disease, which showed mixed results in previous studies [3, 8]. Fourth, our analysis focused on only one Chinese city, thus, the generalizability of our results is limited. Nonetheless, Yichang is one of only a few cities in China where data from all hospitals are collected in a systematic manner to one database, can ensure the comprehensive, accurate and real-time data of hospitals.

## Conclusions

In conclusion, the present study provides evidence that CO increased total and cause-specific outpatient visits, can increase the risk of RED, CVD, GSD, GID and NPD, especially in the warm seasons. These findings reinforce the importance of ambient CO controls and disease prevention in less polluted areas, and warn the public about the atmospheric CO factors that could impact public health.

## Additional file

Additional file 1: **Table S1.** Associations of daily outpatient visits with ambient air pollutants in six-pollutant models. (DOCX 15 kb)

## Abbreviation

95% CI: 95% confidence interval
CDC: Center for Disease Control and Prevention
CO: Carbon monoxide
CVD: Cardiovascular diseases
df: Degrees of freedom
E-R: Exposure-response
GAM: Generalized additive model
GID: Gastrointestinal diseases
GUD: Genitourinary diseases
ICD: International Classification of Diseases
max: maximal
min: minimal
NO_2_: Nitrogen dioxide
NPD: Neuropsychiatric diseases
O_3_: Ozone
PM_10_: Particles < 10 μm
PM_2.5_: Particles < 2.5 μm
RED: Respiratory diseases
RH: Relative humidity
SD: Standard deviation
SO_2_: Sulfur dioxide
WHO: World Health Organization
