# Supplementary material for: Carbon monoxide and risk of outpatient visits due to cause-specific diseases: a time-series study in Yichang, China
Source: Environ Health. 2019 Apr 23;18:36. doi: 10.1186/s12940-019-0477-3 (PMC6477706; doi:10.1186/s12940-019-0477-3)
Supplement: Supplementary file 1 — Table S1. Associations of daily outpatient visits with ambient air pollutants in six-pollutant models. (DOCX 15 kb) [file 12940_2019_477_MOESM1_ESM.docx]

| **Table S1.** Associations of daily outpatient visits with ambient air pollutants in six-pollutant models | | | | | | |
| --- | --- | --- | --- | --- | --- | --- |
| **Pollutants** | **Total visits** | **RED visits** | **CVD visits** | **GUD visits** | **GID visits** | **NPD visits** |
| CO | **37.77(23.48,52.06)** | **29.16(15.51,42.81)** | **52.54(33.51,71.57)** | **42.25(25.78,58.71)** | **29.82(14.95,44.69)** | **25.15(9.78,40.51)** |
| PM_2.5_ | **-2.78(-4.82,-0.73)** | -1.39(-3.32,0.54) | -2.59(-5.35,0.17) | **-3.89(-6.26,-1.53)** | **-2.28(-4.41,-0.16)** | **-3.09(-5.31,-0.87)** |
| PM_10_ | 0.80(-0.57,2.17) | -0.02(-1.32,1.29) | 0.85(-1.03,2.72) | 1.20(-0.38,2.78) | 0.49(-0.94,1.92) | 0.69(-0.81,2.18) |
| NO_2_ | 2.93(-0.48,6.34) | 2.93(-0.20,6.05) | 2.30(-2.39,7.00) | **5.12(1.11,9.14)** | **4.21(0.65,7.76)** | **4.91(1.19,8.64)** |
| SO_2_ | -3.55(-11.82,4.72) | 5.59(-2.03,13.21) | -9.60(-20.85,1.65) | -7.03(-16.68,2.63) | -8.36(-17.00,0.28) | -4.19(-13.21,4.82) |
| O_3_ | 0.25(-1.00,1.51) | **1.26(0.02,2.50)** | -1.00(-2.72,0.73) | 0.78(-0.65,2.21) | 0.61(-0.69,1.92) | -0.60(-1.96,0.76) |
| **Abbreviation**:  RED, respiratory diseases; CVD, cardiovascular diseases; GUD, genitourinary diseases; GID, gastrointestinal diseases; NPD, neuropsychiatric diseases | | | | | | |
| **Note**: Results was estimated percent increases and its corresponding 95% confidence intervals with 1 mg/m³ increase in CO and 10 µg/m³ increase in PM_2.5_, PM_10_, NO_2_, SO_2_ and O_3_ at lag06 (lag06 was concentration computed as the means of the same and previous 6 days); | | | | | | |
| The statistically significant estimates are highlighted in bold. | | |  |  |  |  |
